# Enhanced primary mental healthcare for Indigenous Australians: service implementation strategies and perspectives of providers

**DOI:** 10.1186/s41256-018-0071-1

**Published:** 2018-06-04

**Authors:** Lennart Reifels, Angela Nicholas, Justine Fletcher, Bridget Bassilios, Kylie King, Shaun Ewen, Jane Pirkis

**Affiliations:** 1 Centre for Mental Health, Melbourne School of Population and Global Health, The University of Melbourne, 207 Bouverie Street, Carlton, VIC 3010 Australia; 2 Melbourne Poche Centre for Indigenous Health, The University of Melbourne, 161 Barry St, Carlton, VIC 3010 Australia

**Keywords:** Indigenous health services, Aboriginal mental health, Mental health services, Health equity, Primary healthcare

## Abstract

**Background:**

Improving access to culturally appropriate mental healthcare has been recognised as a key strategy to address the often greater burden of mental health issues experienced by Indigenous populations. We present data from the evaluation of a national attempt at improving access to culturally appropriate mental healthcare for Indigenous Australians through a mainstream primary mental healthcare program, the Access to Allied Psychological Services program, whilst specifically focusing on the implementation strategies and perspectives of service providers.

**Methods:**

We conducted semi-structured interviews with 31 service providers (primary care agency staff, referrers, and mental health professionals) that were analysed thematically and descriptively.

**Results:**

Agency-level implementation strategies to enhance service access and cultural appropriateness included: the conduct of local service needs assessments; Indigenous stakeholder consultation and partnership development; establishment of clinical governance frameworks; workforce recruitment, clinical/cultural training and supervision; stakeholder and referrer education; and service co-location at Indigenous health organisations. Dedicated provider-level strategies to ensure the cultural appropriateness of services were primarily aimed at the context and process of delivery (involving, flexible referral pathways, suitable locations, adaptation of client engagement and service feedback processes) and, to a lesser extent, the nature and content of interventions (provision of culturally adapted therapy).

**Conclusions:**

This study offers insights into key factors underpinning the successful national service implementation approach. Study findings highlight that concerted national attempts to enhance mainstream primary mental healthcare for Indigenous people are critically dependent on effective local agency- and provider-level strategies to optimise the integration, adaptation and broader utility of these services within local Indigenous community and healthcare service contexts. Despite the explicit provider focus, this study was limited by a lack of Indigenous stakeholder perspectives. Key study findings are of direct relevance to inform the future implementation and delivery of culturally appropriate primary mental healthcare programs for Indigenous populations in Australia and internationally.

**Electronic supplementary material:**

The online version of this article (10.1186/s41256-018-0071-1) contains supplementary material, which is available to authorized users.

## Background

Indigenous Australians, who comprise 2.5% of the Australian population, face disproportionally greater burden of mental health issues (including suicidality and mental distress) than non-Indigenous Australians [1–3]. This burden is compounded by significant barriers in Indigenous access to mainstream and specialist mental healthcare services. Known barriers to Indigenous healthcare access in Australia and elsewhere include a lack of trust in health services, non-identification of Indigenous status in health service settings, challenges of service coverage in remote areas, and limited cultural appropriateness of healthcare programs and services [4, 5]. The aim of improving Indigenous access to culturally appropriate mental healthcare services has therefore been identified as a key national health policy strategy to alleviate this mental health burden [6–8].

Two principal avenues for Indigenous people’s access to mental healthcare in Australia exist within the Indigenous-specific and mainstream primary healthcare sectors. The former sector comprises a national network of Aboriginal community controlled health services (ACCHS) which provide a range of healthcare programs, including those underpinned by a framework of social and emotional wellbeing [9]. Nevertheless, nationally 61.5% of Indigenous Australians access ACCHS, while others are likely to be largely reliant on mainstream healthcare services [4].

Healthcare service access and effectiveness have been identified as key components of healthcare quality, with the former centring on service availability, accessibility, affordability and acceptability [10].

While evidence-based models of mental healthcare exist that have proven to be effective in general primary care settings, such as the Australian Access to Allied Psychological Services program [11, 12], these models have only recently become the focus of targeted initiatives, which specifically seek to address the mental healthcare needs of Indigenous people and other ethnic minority groups. A key approach to ensuring both effective and culturally appropriate mental healthcare delivery in such contexts has therefore focused on the cultural adaptation of generic service and treatment models in order to meet the cultural requirements of specific population groups [13].

### Enhanced indigenous primary mental healthcare services

As part of a mental health reform package, two dedicated primary mental healthcare initiatives were introduced through Australia’s national Access to Allied Psychological Services (ATAPS) program. The initiatives incorporated specific enhancements and flexibilities to improve equitable access to and cultural appropriateness of ATAPS services for Indigenous Australians. These enhanced ‘Indigenous ATAPS services’ included the Indigenous mental health service from July 2010 and the Indigenous suicide prevention service from July 2011.

The ATAPS program is a government-funded primary mental healthcare program that facilitates access to appropriate (low or no cost) mental healthcare for people with common mental disorders in the general population [14]. Designed in 2001, the program has been implemented through a national network of 31 regional Primary Health Networks, which formed from a network of 61 primary healthcare organisations (known as Medicare Locals) in July 2015.

Through participation in ATAPS, eligible primary care providers (such as general practitioners, or GPs) can refer consumers with high prevalence disorders (e.g., depression and anxiety) to mental health professionals for up to 12 individual sessions (or 18 in exceptional circumstances) and/or 12 additional group sessions of evidence-based mental healthcare (predominantly cognitive behavioural therapy, or CBT). Referring GPs review treatment after each block of six sessions and upon completion.

Table 1 summarises key features and flexibilities of enhanced Indigenous ATAPS services by contrast to the general ATAPS initiative, with a detailed outline available elsewhere [15–17].

**Table 1:** Key features of enhanced Indigenous ATAPS services and the general ATAPS initiative

Key feature	General ATAPS	Indigenous Mental Health Service	Indigenous Suicide Prevention Service
First introduced	July 2001	July 2010	July 2011
Primary target group	Australians with high prevalence disorders of mild to moderate severity managed in primary care	Indigenous Australians with high prevalence disorders of mild to moderate severity managed in primary care	Indigenous Australians at moderate risk of suicide or self-harm managed in primary care^a^
Mandatory initiative for Medicare Locals	Yes	Yes	No
Eligible referrers	• GP • Psychiatrist • Mental health professional • Paediatrician	• GP • Psychiatrist • Mental health professional^b^ • Paediatrician^b^ • Indigenous health organisation / Aboriginal community controlled health service^b^	• GP • Mental health professional • Paediatrician • Emergency department • Hospital ward • Indigenous health organisation / Aboriginal community controlled health service • Drug and alcohol service • Acute mental health team
Annual session limit	6–12 (18 in exceptional circumstances) individual and 12 group sessions	6–12 (18 in exceptional circumstances) individual and 12 group sessions	Unlimited individual sessions during three month period
Other flexibilities	• Low or no cost to client	• Low or no cost to client • Introduced narrative therapy as a permissible ATAPS intervention • New outreach modality • Sessions can involve the whole family	• Low or no cost to client • No diagnosis or GP mental health treatment plan required at referral • Sessions can involve the whole family • Clinician contacts client within 24 h and provides first session within 72 h of referral (or earlier, as required)

*Acronyms*: *ATAPS* Access to Allied Psychological Services, *GP* General Practitioner

*Note*: Key features as of June 2015. a Not suitable for individuals at acute risk, or with recurrent thoughts, of suicide or self-harm who are more appropriately managed by state and territory mental health services. b Provisional referrals require a GP or psychiatrist prepared mental health treatment plan ideally within 2 weeks of the first session, or as soon as practical

The primary aim of the Indigenous mental health service is to provide Indigenous Australians with an ‘increased level of access to evidence based short-term focused psychological strategies services that are culturally appropriate, within a primary care setting’ [15]. The service is designed for Indigenous people with, or at risk of developing, a mild to moderate mental disorder, and who are referred to the service through their treating GP or psychiatrist, or via provisional referral from a mental health professional, Indigenous health organisation, ACCHS, or paediatrician.

The Indigenous suicide prevention service provides priority ATAPS access to Indigenous Australians who have attempted, or are at moderate risk of, suicide, family members and friends of those who have died by suicide and are at risk themselves, and other Indigenous people with mental health problems at risk of suicide or self-harm [16]. Eligible clients access the service through a GP referral, or via provisional referral from an emergency department, hospital ward, acute mental health team, Indigenous health organisation, ACCHS, paediatrician, ATAPS mental health professional, or drug and alcohol service.

Recent research has shown that the introduction of enhanced Indigenous ATAPS services resulted in substantial increases in Indigenous mental healthcare access over a relatively short period of time, as evidenced by an eightfold increase in the uptake of Indigenous ATAPS services and associated doubling in overall ATAPS program uptake by Indigenous people between 2010 and 11 and 2012–13 [17].

Yet despite these evident gains in service uptake, little is known about key factors that were underpinning and driving the successful national implementation of Indigenous ATAPS services. This qualitative exploratory study therefore utilised semi-structured interviews to examine the service implementation strategies and perspectives of providers involved in a national effort to enhance primary mental healthcare for Indigenous Australians through the ATAPS program. Such research has the potential to inform the future implementation and delivery of culturally appropriate primary mental healthcare services for Indigenous people in Australia and internationally.

## Methods

### Sampling and recruitment

Key informants involved in the implementation of Indigenous ATAPS services, including administering Medicare Local staff, referrers, and mental health professionals were targeted for participation in semi-structured telephone interviews. An initial sample of 19 Medicare Locals was selected purposively to represent a broad cross-section of: all Australian states and territories; metropolitan, regional, rural and remote areas; agencies with recorded referrals for the Indigenous mental health and/or Indigenous suicide prevention service; and agencies experiencing particularly high or low service uptake.

Sixteen ATAPS administering staff from Medicare Locals providing Indigenous ATAPS services agreed to participate in an interview. Two further Medicare Locals facilitated recruitment of representatives from external provider agencies that had been subcontracted by the Medicare Local to deliver these services. One Medicare Local did not respond to the study invitation. Twelve Medicare Locals were asked to act as intermediaries in the recruitment of at least one referrer (preferably from an ACCHS, Acute Mental Health Service, or a GP) and 11 Medicare Locals were asked to facilitate recruitment of mental health professionals delivering Indigenous ATAPS services. All interviewees were contacted by telephone and/or email and followed up by telephone as required. As a secondary recruitment strategy for ACCHS, four ACCHS were followed up directly by the researchers with agreement of Medicare Locals. Time constraints associated with the research precluded more extensive recruitment. All participants provided written consent to participate in the study. The study protocol was approved by the Human Research Ethics Committee of the University of Melbourne.

### Data collection

Three researchers (LR, AN, JF) conducted 31 semi-structured telephone interviews between November 2013 and January 2014, using interview schedules. The interviews focussed on key strategies and issues related to the implementation of Indigenous ATAPS services, such as in regard to: service establishment; stakeholder engagement and service partnerships; service demand, capacity and coverage; the referral process; mental health professional involvement; quality assurance and clinical governance; cultural adaptation and appropriateness; service impacts; implementation barriers and facilitators; and service improvement strategies. Key interview domains and associated data sources are outlined in Table 2. Custom-designed interview schedules comprised a combination of both open-ended questions (to gather qualitative participant feedback) and closed questions with prespecified response options (or Likert-style rating scales) to enable the quantification of responses (such as in relation to local service demand and capacity). Further detail on the interview schedules is available from the authors. The average interview duration for ATAPS administering agency staff, referrers, and professionals was 42, 21 and 30 min, respectively. All interviews were conducted in English, audio-recorded digitally and transcribed through a professional transcription service.

**Table 2:** Key interview domains and data sources

Key Interview Domain	Interview Data Sources		
	Agency staff^a^ *n* = 18	Referrers^b^ *n* = 5	Professionals^c^ *n* = 8
Service establishment	x		
Stakeholder engagement and service partnerships	x	x	
Service demand, capacity and coverage	x	x	x
Referral process		x	x
Mental health professional involvement	x		x
Quality assurance and clinical governance	x		
Cultural adaptation and appropriateness	x	x	x
Service impacts (on clients and referrers)		x	x
Implementation barriers and facilitators	x	x	x
Service improvement strategies	x	x	x

^a^includes ATAPS administering agency staff of Medicare Locals and subcontracted provider agencies

^b^includes eligible ATAPS referrers

^c^includes eligible mental health professionals delivering ATAPS

### Data analyses

Study data were analysed through a combination of thematic and descriptive analyses. Qualitative interview data were examined through thematic analysis [18], following a realist paradigm to report on the experiences, meanings and reality of participants, and using NVivo v10. Initial a priori codes were developed based on broad content domains of interview questions. Two doctoral-level Research Fellows (LR, AN) then coded all transcript data against these. Subsequently, semantic themes were derived inductively from the interview data coded underneath each of the a priori codes. Finally, resulting themes were reviewed in iterative and comparative ways to identify patterns and potential relationships between events and general processes. While we did not formally assess inter-coder reliability, all remaining disagreements in the coding of participant responses between the two coders were resolved by way of consensus and through further clarification of the coding scheme. Data saturation was reached regarding agency staff interviews, but not in relation to mental health professional and referrer interviews. Wherever possible, key themes were therefore corroborated across the three participant groups to enhance the validity of study findings. Additional file 1: Table S1 shows the resulting thematic coding framework for Medicare Local staff as the largest participant group. Quantifiable interview data were examined through descriptive analyses conducted in SPSS v22.

## Results

### Sociodemographic participant profile

The 31 study participants included 18 ATAPS administering agency staff (16 from Medicare Locals and two from sub-contracted provider agencies; collectively referred to as ‘agency staff’ in the following), five ATAPS referrers (four GPs and one mental health professional), and eight mental health professionals delivering Indigenous ATAPS services (referred to as ‘professionals’ for brevity). Participants were mostly female (64.5%), between 20 and 64 years of age, and fairly evenly distributed across all Australian states and territories. One interviewee (a Medicare Local staff member) was Aboriginal and six (two referring GPs and four professionals) provided services from within Indigenous organisations. All interviewed professionals, including seven psychologists and one occupational therapist, held full professional registration and seven had prior experience providing mental health services to Indigenous clients. Additional file 2: Tables S2 outlines the sociodemographic participant profile.

All participating agencies had delivered the Indigenous mental health service (for a mean of 20.4, range 5–41, months) and five had delivered the Indigenous suicide prevention service (mean 14.8, range 5–23, months). Agency staff roles included program manager (13), project officer (1) and other roles (4). All five referrers had been involved in the Indigenous mental health service (mean 8.8, range 3–23, months), referring 22 (range 2–50) clients on average; but not the Indigenous suicide prevention service. Seven professionals had delivered the Indigenous mental health service (mean 25.3, range 4–47, months) and two the Indigenous suicide prevention service (mean 31.0, range 15–47, months). On average, professionals had seen 91 (range 0–374) clients and delivered 314 (range 0–1320) sessions through the former, and 9 (range 1–16) clients and 29 (range 2–56) sessions, respectively, through the latter, service.

The 18 participating agencies represented 94.7% of the 19 agencies originally targeted for participation in this study, and 29.5% of all 61 existing Medicare Locals. The combined service volume of participating agencies accounted for 50.5% of all national Indigenous mental health service, and 77.8% of all national Indigenous suicide prevention service, sessions delivered through the ATAPS program at the time of this study. Interviewee responses referring to both Indigenous ATAPS services are summarised in the following, with illustrative quotes provided.

### Service establishment

Strategies adopted by agencies to determine the local need for Indigenous ATAPS services included: consultation with local Indigenous and non-Indigenous stakeholders, conducting demographic population research, service mapping or gap analysis, needs assessment, and utilising Indigenous ATAPS advisory group members, client feedback, and ATAPS service data.

Most agencies built on existing ATAPS initiatives in order to establish Indigenous ATAPS services. Specific implementation steps involved stakeholder service promotion; co-location of service and professionals at ACCHS; development of ACCHS referral pathways; ensuring adequate professional qualification, registration, experience and training; procurement of provider agencies; amending databases and referral forms; extending the existing triage role; appointing a dedicated project officer; interpreting guidelines; and establishing clinical governance processes.

‘we sort of incorporated it into our existing ATAPS service. So we worked in collaboration with our Aboriginal primary healthcare team here at the Medicare Local to raise awareness about the funding in this area and our capacity to see Aboriginal and Torres Strait Islander clients and the appropriate referral pathways in. And we ensured that we had mental health professionals who had completed appropriate cultural competency training to see Indigenous clients.’ *(ML04, Female, Medicare Local staff)*

‘what we did in 2012 was change the model. And instead of having two dedicated workers to suicide prevention, we put some suicide prevention FTE [full-time equivalent] into all of the mental health workers. So we gave them all 0.2 FTE to supervise the Indigenous suicide prevention service. And that meant the people that were already employed were already going to the community, which meant that at the service we weren’t doubling up on costs of accommodation and travel. But it also meant that people who were already in our general mental health counselling service who needed Indigenous suicide prevention could stay with the same worker.’ *(ML05, Male, Provider agency staff)*

### Stakeholder engagement and service partnerships

Eight agencies had established some form of working partnership with an ACCHS. Other Indigenous partners engaged included district and community health services, health and wellness centres, hospitals, health network, maternal health service, mental health service, GP clinic, and non-health agencies, Indigenous peak bodies, and schools with strong Indigenous representation.

Strategies to develop and maintain these partnerships included establishment of formal linkages (e.g., memorandum of understanding, advisory group, utilising existing networks and forums), the facilitative role of ‘Closing the Gap’ teams, active grassroots engagement with Indigenous communities, longstanding relationships with Indigenous organisations, and to a lesser extent involvement of the Aboriginal primary healthcare team, Indigenous coordinator, senior management, and direct contact with or provision of written information to external staff.

Most agencies reported well established relationships with relevant non-Indigenous service partners, including hospitals and emergency departments, GPs, health services and networks, and less so with public mental health or, alcohol and substance abuse services, NGOs, paediatricians, psychiatrists, welfare and housing agencies, and the Department of Human Services.

Ten agencies had encountered some difficulty establishing external service partnerships, which impeded effective local service implementation. Such difficulties involved the establishment of relationships with the ACCHS (due to ACCHS staff turnover, or mistrust of non-Indigenous organisations), uncertainties of primary healthcare reforms, failure to co-locate the service within a GP clinic, GP turnover, and relationships with state mental health or district health services (due to heavy service workload, a silo mentality, or lacking role clarity). Strategies employed to overcome such difficulties includes time, persistence and making the right connection; a shared focus on client outcomes; and sourcing alternative office space.

‘persistence really would be the main thing if you are having difficulty with a partnership, the recent progress that we have made partnering with our local Aboriginal health service is just because we have had a new team member reach out to them since she has joined the Medicare Local. Sometimes it will be that you just make the right connection with a particular person within that other organisation it can make all the difference.’ *(ML04, Female, Medicare Local staff)*

Most agency staff reported an overall positive stakeholder response to the introduction of Indigenous ATAPS services, with only one reporting a mixed and another a disappointing response.

‘It’s been positive, I think to begin with there was some suspicion about whether the service was really going to be culturally appropriate. In the end, certainly with GPs, the [state health department], those sorts of community organisations they were really open and very positive about our having that tier [of service]. I think that the people that were the most difficult to engage were people from the Aboriginal community up here, but as I say, they have improved considerably.’ *(ML08, Female, Provider agency staff)*

‘I think it’s been extremely positive. Our ability to increase service provision in specifically some of the more rural and remote locations has been very greatly welcomed.’ *(ML12, Male, Medicare Local staff)*

### Service demand, capacity and coverage

Agency staff reported overall considerable but locally variable levels of Indigenous ATAPS services demand, with ratings of ‘little’ (5), ‘moderate’ (7), ‘high’ (2), or ‘very high’ (4) demand on a five-point scale from ‘no’ to ‘very high’ demand. Similarly, referrers reported ‘little’ (1), ‘high’ (1) or ‘very high’ (3) demand. Six professionals indicated that Indigenous ATAPS services had filled a local service gap, whereas two did not.

All interviewees indicated at least a moderate level of capacity to meet existing service demand. Staff rated agencies ‘moderately’ (4), ‘mostly’ (7), or ‘completely’ (7) able to meet demand (on a five-point scale from ‘not at all’ to ‘completely’). Referrers found that service demand had been ‘moderately’ (1), ‘mostly’ (2), or ‘completely’ (2) met.

Most interviewed agency staff identified hard to target subgroups who could potentially benefit from Indigenous ATAPS services, including: those not engaged with Aboriginal medical services, adolescents, people in remote or rural areas, homeless and transient groups, those requiring home visits or transport, those with more complex issues or not self-identifying in mainstream general practice, females, Indigenous clients in general, and NGO clients.

‘those that don’t engage with the AMS [Aboriginal Medical Service], that don’t feel the AMS is for them, they are really the population that we are trying to engage, and are difficult to engage. If they're not engaged with the AMS, they're usually not engaged with another GP, because of the cost and lack of bulk billing, so it’s difficult to then get that care plan and referral.’ *(ML02, Female, Medicare Local staff)*

‘the transient populations that do come down from the lands that don’t stay in the place where our service is located for any length of time. I think that’s the problem with most of our mental health services. You know, you have got levels of outreach but there is still that hub-spoke approach.’ *(ML13, Male, Medicare Local staff)*

Specific factors identified as impacting on lower service coverage for these groups included workforce issues (e.g., lack of Indigenous mental health workers and liaison officers), limited ATAPS model flexibility, access to bulk billing GPs, or facilities. Other factors impeding greater service coverage included locational distance or travel required, funding limitations (e.g., to provide outreach services), reluctance to engage with health services, stigma surrounding mental health treatment, the need for confidentiality in small communities, lacking service awareness, service coordination for transient groups, availability of another service, variable family support, cultural gender roles, and other access issues.

‘if we had a liaison officer who could go out to every single community and find out what the needs were, we’d get to know more about the needs, if there are people who would benefit from ATAPS who aren’t getting services, but it's hard to get out to every community with limited resource’ *(ML17, Female, Medicare Local staff)*

‘Definitely the requirement to have a GP mental health treatment plan for Indigenous clients is just an ongoing barrier, given that a lot of Indigenous clients are not engaged with a GP. The people that can refer in for Indigenous clients are quite limited; for example, it would be great if there were more people, even within our Medicare Local, that could refer directly to us. It would just take away some of the entry point barriers.’ *(ML04, Female, Medicare Local staff)*

### The referral process

All referrers and one-half of professionals expressed satisfaction with local referral processes, while the other half was either mostly satisfied with some problems or dissatisfied. Referral problems involved varied barriers in the access to GPs, centralised Medicare Local intake resulting in some delay and complexities for clients, the lack of a self-referral option, and occasional referrer confusion about different ATAPS initiatives. All professionals found most or all referrals to be appropriate. Three referrers had received referral education from the Medicare Local (via face-to-face contact, a written information pack, or education evening), one had not, and another directly accessed a professional in that respect.

‘I think sometimes seeing a GP and having a regular GP, for our local population anyway that can be a really big barrier having to get to a GP and talk about mental health issues. I think it’s a general lack of trust in GPs, there’s a lack of Aboriginal GPs, there’s only a few specific Aboriginal mental health services that have GPs and so then also there’s some confidentiality issues because kind of the worrying I guess of people finding out in their community.’ *(MHP06, Female, Mental health professional)*

‘I provide services in another funding stream, and they allow people to self-refer to the service. I get a lot of referrals that way, so I think if ATAPS would allow that you would get more referrals, and then obviously your aim is to link them in with a doctor or a GP or whomever is available in their community, but yeah the referral process is okay.’ *(MHP07, Female, Mental health professional)*

### Mental health professionals

Participating agencies had engaged 187 (mean 10, range 1–30) and 64 (mean 13, range 4–29) professionals, respectively, to deliver Indigenous mental health and suicide prevention services. Professionals were either contracted and/or directly employed; and commonly delivered services in their own rooms, at Medicare Locals, general practices, external agencies, or other locations, such as Indigenous health organisations, hospital, client’s home, or park.

Ten agencies did not utilise Indigenous health workers, eight liaised with Indigenous health workers from other services, and two specifically mentioned the lack of ATAPS relevant skills among Indigenous health workers. Overall, only two Indigenous health workers were involved in direct service delivery, including one in a dual clinical and partnership work role.

‘We don’t [have] an Indigenous health worker. We have our Aboriginal liaison officer and we liaise with our CTG [Closing the Gap] team. What we find is that the guidelines for the Indigenous program, what they're asking of the workers, the Indigenous health workers don’t actually have the counselling skills that are required, and they don’t have the level of training to provide a 12 session CBT-based program to these clients.’ *(ML02, Female, Medicare Local staff)*

‘They are providing 50% clinical services and then 50% sort of partnership work and doing the sort of linkages between the consumer and other services that they may need.’ *(ML16, Female, Medicare Local staff)*

Most non-Indigenous professionals had completed the ATAPS provided cultural competency training, with only four agencies indicating that some and one agency that none, of their non-Indigenous staff had received the training. All professionals delivering Indigenous suicide prevention services had completed ATAPS suicide prevention training. Mean training satisfaction ratings were 8.9 and 6.6, respectively (on a scale from 0 ‘not at all’ to 10 ‘completely’ satisfied).

Most professionals had access to clinical supervision, typically involving individual and/or peer supervision; while contracted professionals were often obliged to organise their own clinical supervision. Professionals from nine agencies had further access to mostly informal cultural supervision through ‘Closing the Gap’ teams, Indigenous organisations, or Aboriginal Elders.

### Quality assurance and clinical governance

Processes established to monitor service quality and ongoing improvement involved service audits and clinical governance arrangements, with some staff citing specific attempts to ensure the cultural appropriateness of clinical governance frameworks. Other quality assurance mechanisms involved: obtaining stakeholder service feedback; monitoring professional qualification, registration and experience; national mental health standards compliance; mental health advisory or reference groups; service benchmarking; quality assurance guidelines; and liaison with Indigenous services.

‘Reviewing policies and procedures to make sure that they are developed with that Indigenous community in mind was a big part of implementing that clinical governance framework. So even our staff recruitment and induction policy and procedure was reviewed to ensure we had Indigenous representation on interview panels and things like that as well.’ *(ML04, Female, Medicare Local staff)*

‘we’re just implementing culturally appropriate feedback forms that our Aboriginal liaison officer has been helping us to implement and to look at ways of being able to ensure we’re getting feedback in a way that is useful to us, but is respectful to the clients as well.’ *(ML02, Female, Medicare Local staff)*

### Cultural adaptation and appropriateness

Agency strategies adopted to ensure the cultural appropriateness of services included cultural awareness training for professionals and staff, consultation with ‘Closing the Gap’ teams or Indigenous workers, enlisting professionals with Indigenous work experience, utilising client feedback, enabling Indigenous service referrals, cultural approval of resources, employing an Aboriginal mental health liaison officer, AMS co-location, matching clients and professionals in terms of gender and experience, and supervision.

‘we made sure that the clinicians have experience dealing with that particular client group, and also that they’ve undertaken the cultural awareness training’ *(ML01, Female, Medicare Local staff)*

‘we also liaise closely with our Closing the Gap team and use them as experts, and link in and engage them when we need support and they also provide us with ongoing education and training’ *(ML02, Female, Medicare Local staff)*

Interviewed professionals commonly provided CBT or narrative therapy interventions, with evidence of some cultural adaptations. Some specifically highlighted the need for a different approach to building rapport with Indigenous clients, which was described as being less direct and probing and as unfolding at a slower pace. Formal and informal Indigenous community involvement was seen as vital to effective client engagement.

‘The first part and in terms of the intervention, I adapt mainstream CBT, particularly acceptance and commitment therapy principles culturally. So, for example, the language I use might be different; instead of using ‘self-talk’ I will often talk about ‘yarning inside your head’. So CBT principles but they’ve got to be culturally adapted and keeping things as concrete as I can, cultural adaptation is really important. Engagement with community with Indigenous clients is, without connection, without relationship they don’t come back, so that initial development of rapport is essential. I allow lots of time for that cultural greeting process, so checking out where somebody comes from, who they might know that I might know, there’s quite a bit of storytelling initially and a little bit of lightness and then, you know, try and allow people to tell their story. I ask guiding questions but in a very different way that I would with non-Indigenous, so I don’t probe and poke too much. Yeah, as you would be aware, the communication style is quite different with Indigenous people.’ *(MHP05, Female, Mental health professional)*

‘I make sure my service is culturally appropriate. I have Indigenous artwork in my office. I have a statement about acknowledging traditional owners and caretakers. I make sure that I attend Indigenous cultural and social events in the community, and I maintain social contact with local Indigenous community members.’ *(MHP08, Female, Mental health professional)*

All referrers rated Indigenous ATAPS services to be ‘very’ (5) culturally appropriate (on a five-point scale from ‘not at all’ to ‘extremely’); while professionals rated them to be ‘moderately’ (4), ‘mostly’ (2), ‘completely’ (1), or ‘minimally’ (1) appropriate for Indigenous clients (on a similar scale). Reasons provided for lower ratings indicated greater scope for funding flexibility, access to cultural mentors or training, home visits, a social and emotional wellbeing focus, Indigenous providers, non-GP referrers, and exclusion of ‘no shows’ from the session limit.

### Service introduction impacts

All five referrers reported positive impacts of the introduction of Indigenous ATAPS services on themselves and their organisation (in terms of the general availability, or perceived greater cultural sensitivity of an Indigenous-specific service, the ability to provide continuing care in the community, increased service access through provider visits, or Medicare Local support received). Four referrers noted positive client impacts in terms of good clinical outcomes, access to Indigenous-specific services, and one-hour appointments. Seven professionals equally noted positive client impacts in terms of clinical outcomes, clients recommending the service to other community members, increased service access and capacity, client willingness to re-engage, the ability to receive care in the community, and attend sessions with other people.

### Implementation barriers and facilitators

Table 3 summarises key barriers and facilitators of service implementation, which are illustrated through quotes in the following. Key facilitating factors for agency staff included good relationships with Indigenous services, and access to experienced professionals, closing the Gap and Indigenous primary healthcare teams:

‘Our ongoing strong relationships and networks with the Aboriginal health services, I think that’s assisted to facilitate that. We already had strong linkages with them and because we had been proactive and already had clinicians in place providing general ATAPS before the Indigenous program came to fruition, I think it’s just naturally evolved from there.’ *(ML10, Male, Medicare Local staff)*

**Table 3:** Service implementation barriers, facilitators and future improvement strategies

	Agency staff^a^	Referrers^b^	Professionals^c^
Implementation barriers	ATAPS model limitations: • Need for GP referral • Accommodating client engagement and complex cases in session limit • Limited capacity to provide outreach or transport • One-on-one model • Challenging timelines in vast areas with transient populations Other barriers: • Establishing Indigenous community or service relationships • Shortage of Indigenous mental health and male professionals • Non AMS clients • No shows • Limited funding • Referrer /AMS turnover • Primary health reform • Non-identification of Indigeneity	• No barriers • Demand management restricting referral • Confidentiality of faxing client referrals	• No shows • Relationship building with Indigenous communities and health services • Limited funding to provide culturally appropriate (outreach) services • Central intake • Limited local/ cultural knowledge of ATAPS Suicide Support Line • Lacking clinical service support • Qualification requirements precluding Indigenous health workers from facilitating CBT-groups • Occasional waiting lists
Implementation facilitators	• Good Indigenous service relationships • Experienced professionals in communities • Indigenous workers from Closing the Gap and Indigenous primary healthcare teams • ATAPS funding • Indigenous ATAPS staff • Good referrer relations • Service demand • Alignment with existing Indigenous services • Willingness to learn how to work with Indigenous people	• Service availability • Streamlined referral • Referrer support • Clinician availability and engagement • Payment for no shows • Lack of a gap payment • Good governance	• Good relationships with Indigenous communities and services • Co-location with Indigenous health services • Good relationships with Medicare Locals • Funding • Project officer role • Skills and experience • Cultural awareness training • Provision of client transport • Flexible interventions • Provisional referral and priority appointments
Future improvement strategies	Cultural appropriateness: • Cultural awareness training and supervision • After-hours suicide line • Outcome measures • Indigenous mental health workers and outreach • Service guidelines Service engagement: • Service promotion and referrer awareness • AMS or NGO linkages • Target AMS non-engaged groups Enhanced service flexibility: • GP referral and treatment plan • Session limit • Session duration • Interventions • Client self-referral • Suicide timelines • Transport allowance Funding: • Non-session time (liaison work) • Rural/remote services • Pool ATAPS funding to flexibly meet demand • Maintain or increase funding Integration and responsiveness: • AMS co-location • Build community capacity to respond to suicidality • Involve Indigenous communities in delivery • Provide mental health first aid training for Indigenous primary care workers	• Increased availability • Client progress and wait list feedback • Referrer engagement • More sessions • Indigenous community engagement • No improvement required	Service flexibility: • Referral pathways (Indigenous health workers, self-referral) • Session limit / duration • Session time / location • Training and supervision funding • Indigenous health workers as providers • No shows • Service access for low income earners • Non-CBT interventions • Diagnosis requirement • Outreach Other strategies: • Cultural supervision / mentoring • Frequent remote visits • Funding increase • Paid consultation time • Direct AMS funding • Provide transport

*Acronyms*: *AMS* Aboriginal Medical Service, *ATAPS* Access to Allied Psychological Services, *CBT* Cognitive behaviour therapy, *GP* General practitioner, *NGO* Non-government organisation

^a^includes ATAPS administering agency staff of Medicare Locals and subcontracted provider agencies

^b^includes eligible ATAPS referrers

^c^includes eligible mental health professionals delivering ATAPS

‘And I think the factors that helped us implement was the fact that we have got a clinician in the Indigenous community in [location] so she is really, really involved in all the NGOs and in the community, very passionate about the community. And she drives everything that we do, if she feels that we don’t do enough she is very vocal about it.’ *(ML03, Female, Medicare Local staff)*

‘I think that it’s been about building that trust with community and for us a lot of that has been as the Closing the Gap team is developing trust and then the Closing the Gap team brings us on board and says that they also trust us.’ *(ML08, Female, Provider agency staff)*

Key agency staff implementation barriers involved perceived ATAPS model limitations and difficulties engaging with Indigenous communities and services:

‘the limitation on sessions is a barrier because oftentimes it takes much longer to even manage engagement with Aboriginal people’ *(ML17, Female, Medicare Local staff)*

‘we are an appointment-based service and we don’t have the ability to go out to people’s homes and often with our population of Aboriginal families that mobility would make a bit of a difference, particularly for those clients that might be suffering with some anxiety and really struggle to access a mainstream type of environment. You know, or have issues using transport or financial issues which will restrict their ability to get places. So yeah, having people come to us, we know that that’s difficult.’ *(ML11, Male, Medicare Local staff)*

‘The fact that we can’t fund a Aboriginal mental health liaison officer position because those people typically can’t do sessions, so we can’t count any activity against their work. So there's many linkages you need in this work, we’ve got diverse Aboriginal communities, they're not all the same, and so there's lots and lots of liaison work that needs to go on that we haven’t actually been able to do effectively.’ *(ML17, Female, Medicare Local staff)*

Referrers had encountered few barriers, while facilitating factors included service availability, streamlined referral, and referrer support. Professionals’ barriers related to client nonattendance and relationship building with Indigenous stakeholders:

‘The main challenge is that in order to build trust in the community as a health provider, you need to be connected to the community, and as a [ATAPS] provider I can’t do that, so I rely on my established network of other workers and people in the community to make the recommendations to someone to come and see me.’ *(MHP08, Female, Mental health professional)*

Key facilitating factors for professionals included good Indigenous community and service relationships, and co-location with Indigenous health services:

‘I’ve been working up here now 5 ½ years. The relationship I have with the remote communities that I go to, when you’ve been around for a while, it facilitates, it makes things easier.’ *(MHP07, Female, Mental health professional)*

‘the ATAPS service being based in the medical service facilitates the provision of Indigenous services, because I can access the health workers over a cup of tea and we’ll have a yarn about what’s been going on in the community. The AMS staff themselves are the best referrers because they know me, they feel safe with me and they refer all their family and friends’ *(MHP05, Female, Mental health professional)*

### Future improvement strategies

Improvement strategies (Table 3) of agency staff were partly aimed at enhancing future service cultural appropriateness (e.g., through cultural supervision of staff; cross-service appointment of Indigenous mental health workers) and engagement (e.g., via targeted service promotion).

‘Recruit an Aboriginal mental health worker to work across ATAPS and the Aboriginal primary healthcare team and to look into the option of doing some outreach work to increase our accessibility to Aboriginal community.’ *(ML04, Female, Medicare Local staff)*

‘look at how we’re targeting our marketing and promotion as a program, to make sure that we’re really aiming at those groups that probably aren’t engaged with the AMS’ *(ML14, Female, Medicare Local staff)*

Other agency staff strategies were geared to enhance program flexibility (e.g., increasing the session limit to cater for complex client presentations; exploring alternatives to GP intake), associated funding models (to reflect vital liaison work; the complexity of client issues; costs of rural and remote service delivery; flexible pooled funding to meet presenting service demand), as well as service integration/responsiveness (e.g., via AMS co-location).

‘taking away the barriers that really make the ATAPS services rigid and not accessible. And that is the requirement to have a GP upfront and a mental health treatment plan up front. It should remain as part of the journey, not as a prerequisite.’ *(ML03, Male, Medicare Local staff)*

‘it's sometimes good to have liaison officers, so some acknowledgement that we can employ someone who doesn’t do sessions but does a whole lot of the connection stuff, the connection work between communities, Aboriginal people, Aboriginal organisations and us.’ *(ML17, Female, Medicare Local staff)*

‘Co-locating within the AMS for a component of time would be really worthwhile to make the service more readily available’ *(ML14, Female, Medicare Local staff)*

Referrers primarily sought to improve ongoing service availability, feedback and engagement:

‘it would be great if the psychologist could somehow let the reception know that the appointment’s been made you know for peace of mind for me’ *(REF01, Female, Referrer)*

Professionals equally highlighted future avenues for and benefits of greater service flexibility regarding referral pathways, session format, funding use, and Indigenous health worker involvement.

‘It could entail referral from Indigenous health workers or self-referral.’ *(MHP05, Female, Mental health professional)*

‘And for the funding to be a bit more flexible in how we use it, whether that’s on outreach workers or staff training and ongoing supervision’ *(MHP06, Female, Mental health professional)*

‘An Aboriginal person with a mental health qualification could be a provider of ATAPS services, rather than it having to come from a psychologist, mental health nurse or social worker’ *(MHP08, Female, Mental health professional)*

## Discussion

Designed to facilitate collaboration between Indigenous and mainstream healthcare sectors, Indigenous ATAPS services provide an avenue for access to appropriate mental healthcare for Indigenous Australians who may or may not be engaged with the ACCHS [9]. As Primary Health Networks and provider agencies move to establish such services and navigate their place in the Indigenous mental health space, no single recipe for successful implementation is likely to do justice to the diversity of local Indigenous community scenarios and healthcare service landscapes. Yet, provider interview data highlighted a number of key strategies and ingredients that are likely to prove critical in such implementation attempts.

Building on existing program flexibilities, such as provisional referral pathways and flexible session modalities (Table 1), agency-level implementation strategies to enhance service access and cultural appropriateness included: the conduct of local service needs assessments; Indigenous stakeholder consultation and partnership development; establishment of clinical governance frameworks; workforce recruitment, clinical/cultural training and supervision; stakeholder and referrer education; and service co-location at Indigenous health organisations.

Dedicated provider strategies to ensure the cultural appropriateness of services were primarily aimed at the context and process of delivery (involving, flexible referral pathways, suitable locations, adaptation of client engagement, and service feedback processes) and, to a lesser extent, the nature and content of interventions (provision of culturally adapted CBT or narrative therapy) [19]. Notwithstanding the lack of an explicit Indigenous stakeholder perspective as the ultimate arbiter of successful implementation, most interviewed providers deemed the services to be culturally appropriate. Overall, this finding appeared to be corroborated by substantial resulting increases in service uptake by Indigenous consumers [17].

Nevertheless, existing interview data (in lieu of a designated Indigenous stakeholder perspective) are insufficient to ascertain the cultural appropriateness of Indigenous ATAPS services. Moreover, the adaptation of mainstream primary care services provides but one avenue to addressing the existing burden of mental health issues among Indigenous populations [20]. It has further been argued that attempts at fostering the cultural appropriateness of services and cultural competency of providers may inadvertently run the risk of reifying cultural stereotypes and producing racialized accounts of health [21]. Such orientation can therefore detract from the broader complex array of historical and social issues and structural inequalities that often underpin presenting mental health problems, and which may be difficult to address by means of psychological therapy alone, even if culturally adapted. Beyond intercultural competency, Indigenous healthcare providers may therefore benefit from acquiring greater ‘structural competency’ in terms of an awareness of the broader social structures and institutional forces that influence Indigenous health above the individual level [22]. In view of the initial challenges encountered by some local agencies in establishing trusting Indigenous stakeholder relationships, such structural competency can therefore also be instrumental in fostering truly transformative partnerships between Indigenous and mainstream healthcare services [23].

Key ingredients facilitating service implementation beyond funding availability included local knowledge; integration within the existing foundation of other ATAPS initiatives; criticality of trust and good Indigenous client, community and stakeholder relations; experienced professionals; and close collaboration with Indigenous healthcare services (such as Bridging the Gap and Aboriginal Primary Healthcare teams).

Despite adopted strategies, providers faced varied implementation challenges. These concerned ATAPS model limitations (e.g., accommodating more complex client presentations and engagement work in the session limit, referral pathway barriers, need to see a GP); restricted outreach or client transport capacity; hard to target Indigenous subgroups (e.g., rural and remote, not GP or ACCHS engaged); and limited Indigenous health worker involvement.

While suitably qualified Indigenous health professionals were eligible to provide Indigenous ATAPS services, broader Indigenous health workforce shortages [24] and specific ATAPS-relevant skill shortages among Indigenous health workers (vis-a-vis existing guideline requirements) may have limited ATAPS involvement. These point to a continued need for Indigenous health workforce development and to the importance of flexible avenues for the involvement of Indigenous professionals in the delivery of enhanced primary mental healthcare services. Since ATAPS guidelines were flexibly operationalised by agencies, perceived ATAPS model limitations (e.g., regarding referral pathways) may have reflected specific local service arrangements, limited provider awareness of existing flexibilities, or broader program limitations (such as the annual session limit or referral-based intake and service model).

Broadly speaking, implementation of the referral-based ATAPS service model tends to work well where eligible treatment-coordinating referrers (i.e., GPs and psychiatrists) exist [25]. However, not unlike similar services, it can face challenges in rural and remote locations where access to these professionals rapidly diminishes, or where GP visits may not constitute ‘culturally normalised’ activities. Nonetheless, the evidence suggests that numerous agencies successfully utilised their outreach capacity to provide Indigenous ATAPS services in rural and remote communities. Frequently, such efforts relied on experienced professionals engaged with Indigenous communities and close agency collaboration with Indigenous healthcare services. If Indigenous clients in remote areas are to be increasingly targeted in the future, outreach, transport and flexible modes of service delivery are likely to become focal issues that will need to be addressed. While stronger service integration in Indigenous healthcare may be a desirable goal and flexible service delivery modes already exist within ATAPS, efficient outreach and Indigenous community engagement efforts are currently frequently reliant on ATAPS-external service capacities.

### Recommendations

Our study findings indicate that innovations in primary mental healthcare program design [25] aimed at enhancing Indigenous service access and cultural appropriateness need to be complemented with dedicated local agency and provider-level efforts in partnership with Indigenous stakeholders to optimise the integration, adaptation and utility of these services within local Indigenous community and healthcare service contexts.

Taken together, service provider perspectives indicate that future implementation and utility of Indigenous ATAPS services could be further improved through: 1) enhanced guideline flexibility (in terms of Indigenous health worker or client self-referral, and the session limit); 2) increased provider and stakeholder awareness of existing program flexibilities; 3) stronger recognition of vital Indigenous community and stakeholder engagement work; 4) smarter service provision strategies (e.g., service collaboration, co-location, capacity integration, hybrid service delivery/liaison roles, cross-service appointments, or subcontracting ACCHS as provider agencies); 5) strengthening outreach capacity; 6) continuous workforce development (both of Indigenous workers and upskilling of non-Indigenous workers in socio-cultural and structural competency); and 7) cultural supervision.

Several of these recommendations resonate with the wider literature, which highlights the importance of appropriate cultural engagement, informal referral processes, dedicated outreach, flexible service delivery models, and dual clinical/cultural provider competence and supervision as vital means to facilitating Indigenous healthcare service access and engagement [26–28]. They are also of particular significance in view of flexible pooled funding arrangements for psychological therapy services which are to be introduced in Australian primary mental healthcare from July 2016.

### Limitations

Despite the explicit provider focus and substantial service volume accounted for by participating agencies, this study was limited by a lack of data on the perspectives of Indigenous stakeholders, referrers and mental health professionals. The lack of a designated Indigenous stakeholder and service user voice impeded the examination of the ultimate service implementation success and cultural appropriateness from an Indigenous perspective. Study findings are therefore primarily reflective of the perspectives of service providers. Having said that, one member of our research team (SE) a senior Indigenous academic provided an invaluable Indigenous perspective on the interpretation of study results and broader contextualisation of findings. It is further conceivable (although not directly evident) that the service evaluation intention of the study, combined with the academic background and largely non-Indigenous composition of the study team may have hampered more effective Indigenous stakeholder engagement. Data collection via telephone interviews put inevitable limits on the ability to capture non-verbal aspects of participant communication.

## Conclusions

National efforts to enhance mainstream primary mental healthcare services for Indigenous populations are critically dependent on effective local agency- and provider-level strategies to optimise the integration, adaptation and utility of these services within local Indigenous community and healthcare service contexts. Despite its explicit provider focus, this study was limited by a lack of Indigenous stakeholder perspectives. Key study findings are of relevance to inform the future implementation and delivery of culturally appropriate primary mental healthcare programs for Indigenous populations in Australia and internationally.

## Additional files

Additional file 1: **Table S1.** Thematic Coding Framework (Medicare Local Staff). (DOCX 19 kb)

Additional file 2: **Table S2.** Sociodemographic participant profile (*N* = 31). (DOCX 21 kb)
