# Supplementary material for: Enhanced primary mental healthcare for Indigenous Australians: service implementation strategies and perspectives of providers
Source: Glob Health Res Policy. 2018 Jun 4;3:16. doi: 10.1186/s41256-018-0071-1 (PMC5985563; doi:10.1186/s41256-018-0071-1)
Supplement: Supplementary file 1 — Table S1. Thematic Coding Framework (Medicare Local Staff). (DOCX 19 kb) [file 41256_2018_71_MOESM1_ESM.docx]

**Table S1** Thematic Coding Framework (Medicare Local Staff)

| **Interview Domain**  **(A priory code)** | **1^st^ Level Theme**  **(Semantic)** | **2^nd^ Level Theme**  **(Semantic)** |
| --- | --- | --- |
| Service establishment | ML establishment steps / work | Appoint project officer  Change referral form or data base*  Extend existing triage role  Continue already established services*  Develop referral pathways through AMS*  Ensure MHP qualification, experience, training*  Establish clinical governance  Establish service at AMS  Interpret guidelines  Procure provider agency  Service promotion |
|  | Determining local service need | Client feedback  Stakeholder consultation  ATSI consumer representative on ATAPS reference group  Demographic research  Looking at data from ATAPS Minimum Data Set  Needs assessment  Service mapping / gap analysis |
| Stakeholder engagement and service partnerships | Stakeholder response | Overall positive*  Mixed response  Disappointing |
|  |  |  |
|  | Indigenous service partnerships | ACCHS & AMS*  GP Clinics with Indigenous focus  Other Indigenous organisations* |
|  |  |  |
|  | Develop/maintain partnerships | Aboriginal coordinator  Aboriginal primary health team facilitates  Active grassroots engagement with community  Attendance at networks and forums  Contact with staff of services and referrers  CTG facilitates  Formal linkages (MOU, advisory group)  Longstanding relationship  Provide program information  Senior management takes responsibility |
|  |  |  |
|  | Other service partnerships  Challenges establishing partnerships  How challenges were overcome | Well established*  Not yet established  Established through other programs  Building relationship with AMS  Negotiating co-location with GP clinic  Engagement with state mental health  Establishing services within health district  GP turnover / referrer education  Primary health reform  Time, persistence, making the right connection  Educate GPs and practice managers  Putting the client first - focus on outcomes  Not overcome - found alternative |
|  |  |  |
| Service demand, capacity and coverage | Hard-to-target subgroups | Adolescents  Females  Homeless people  NGO clients  Rural population groups  People in remote areas  People requiring home visits  Those not self-identifying in general practice |
|  |  | People with complex issues |
|  |  | People with transport issues  Those not engaged with the AMS  Transient populations  Whole ATSI population |
|  | Key factors impacting on lower treatment coverage | Access to bulk billing GPs  Access to rooms  ATAPS model lacks flexibility  Awareness of service  Non-disclosure of Indigeneity in mainstream service  Confidentiality in small communities  Cultural issues  Finding staff accommodation  Funding  Location distance  Travel  Other services available  Reluctance to engage with health services  Service access  Service coordination  Stigma of mental health treatment  Variable family support  Workforce issues |
|  | Strategies to improve service delivery and treatment coverage | AMS co-location  Creative interpretation of guidelines  Employing Aboriginal liaison officer  Engaging referral partners*  Enhance ATAPS model flexibility*  Identifying ATSI clients  Improving service coordination  Liaising with CTG team  More funding*  Open dialogue to identify best treatment model for remote communities*  Outreach work  Service promotion in community* |
| Referral process | *Not captured in ML interviews* | *Not captured in ML interviews* |
| Mental health professionals | Utilisation of ATSI health workers  Clinical and cultural MHP supervision arrangements | ATSI health workers lacking mental health skills  Liaising with other ATSI workers (outside of ATAPS)  Dual clinical / partnership work role  Clinical*  Clinical and cultural combined*  Cultural* |
| Quality assurance and clinical governance | Key mechanisms | Auditing*  Benchmarking  Clinical governance*  Developing quality assurance guidelines  Compliance with national mental health standards*  Meeting with Indigenous services  Mental health advisory / reference groups  Mental health professionals*  Respond to feedback* |
| Cultural adaptation and appropriateness | Steps taken to ensure cultural appropriateness | Aboriginal mental health liaison officer  Consultation with CTG and ATSI worker  Co-location with AMS  Cultural awareness training*  Match client to MHP (gender, experience)  MHPs with ATSI work experience  Appropriate referrals from ATSI services*  Cultural approval of resources  Respond to client feedback  Supervision and peer supervision |
| Service impacts | *Not captured in ML interviews* | *Not captured in ML interviews* |
| Implementation barriers and facilitators | Facilitating Factors  Implementation Barriers | Good Indigenous service relationships  Experienced professionals integrated in communities  Indigenous staff from CTG and primary health teams  ATAPS funding  Indigenous ATAPS staff  Good referrer relations  Service demand  Alignment with existing Indigenous services  Physical clustering of services (co-location)  Willingness to learn how to work with Indigenous people  ATAPS model limitations*  Establishing Indigenous community or service relationships*  Workforce challenges*  Non AMS clients  Client no shows*  Limited funding  Primary health reform  Non-identification of Indigeneity |
| Service improvement strategies | Cultural appropriateness  Service engagement  Service flexibility  Funding  Integration / responsiveness | Cultural awareness training and supervision  After-hours suicide line  Outcome measures  Indigenous mental health workers and outreach  Service guidelines  Service promotion and referrer awareness  AMS or NGO linkages  Target groups not engaged with AMS  GP referral and treatment plan  Session limit  Session duration  Interventions  Client self-referral  Suicide prevention timelines  Transport allowance  Non-session time (liaison work)  Rural/remote services  Pool ATAPS funding to flexibly meet demand  Maintain or increase funding  AMS co-location*  Build community capacity to respond to suicidality  Involve Indigenous communities in delivery  Mental health first aid training for Indigenous primary care workers |

Acronyms: ATAPS - Access to Allied Psychological Services, AMS - Aboriginal Medical Service, ATSI - Aboriginal and Torres Strait Islander, CTG - Closing the Gap, GP - General Practitioner, MHP - Mental health professional, ML - Medicare Local, MOU - Memorandum of Understanding, NGO - Non-government organisation.

Note. Due to space limitations, 3^rd^ Level themes are not listed in the table (‘*’ denotes 2^nd^ level themes with additional 3^rd^ Level subthemes). While the above thematic coding framework underpinned the qualitative analysis of open-ended interview questions, interview schedules also contained several questions with pre-specified response options to gather quantitative information regarding these content domains.
