# Supplementary material for: Enhanced primary mental healthcare for Indigenous Australians: service implementation strategies and perspectives of providers
Source: Glob Health Res Policy. 2018 Jun 4;3:16. doi: 10.1186/s41256-018-0071-1 (PMC5985563; doi:10.1186/s41256-018-0071-1)
Supplement: Supplementary file 2 — Table S2. Sociodemographic participant profile (N = 31). (DOCX 21 kb) [file 41256_2018_71_MOESM2_ESM.docx]

**Table S2** Sociodemographic participant profile (N=31)

|  | Agency staff^a^ | | | Referrers^b^ | | | | Professionals^c^ | | |
| --- | --- | --- | --- | --- | --- | --- | --- | --- | --- | --- |
|  | **No.** | | **%** | **No.** | | **%** | | **No.** | | **%** |
| Gender | | | | | | | | | | |
| Female | | 11 | 61.1 | 2 | 40.0 | | 7 | | 87.5 | |
| Male | | 7 | 38.9 | 3 | 60.0 | | 1 | | 12.5 | |
| Total | | 18 | 100.0 | 5 | 100.0 | | 8 | | 100.0 | |
| Indigeneity | | | | | | | | | | |
| Aboriginal | 1 | | 5.6 | 0 | 0.0 | | | 0 | 0.0 | |
| Torres Strait Islander | 0 | | 0.0 | 0 | 0.0 | | | 0 | 0.0 | |
| Both | 0 | | 0.0 | 0 | 0.0 | | | 0 | 0.0 | |
| Neither | 17 | | 94.4 | 5 | 100.0 | | | 8 | 100.0 | |
| Total | 18 | | 100.0 | 5 | 100.0 | | | 8 | 100.0 | |
| Age range |  | |  |  |  | | |  |  | |
| 20-24 | 1 | | 5.6 | 0 | 0.0 | | | 0 | 0.0 | |
| 25-29 | 0 | | 0.0 | 1 | 20.0 | | | 2 | 25.0 | |
| 30-34 | 3 | | 16.7 | 1 | 20.0 | | | 1 | 12.5 | |
| 35-39 | 4 | | 22.2 | 1 | 20.0 | | | 1 | 12.5 | |
| 40-44 | 1 | | 5.6 | 1 | 20.0 | | | 1 | 12.5 | |
| 45-49 | 4 | | 22.2 | 0 | 0.0 | | | 1 | 12.5 | |
| 50-54 | 4 | | 22.2 | 0 | 0.0 | | | 1 | 12.5 | |
| 55-59 | 0 | | 0.0 | 1 | 20.0 | | | 1 | 12.5 | |
| 60-64 | 1 | | 5.6 | 0 | 0.0 | | | 0 | 0.0 | |
| 65-69 | 0 | | 0.0 | 0 | 0.0 | | | 0 | 0.0 | |
| 70-74 | 0 | | 0.0 | 0 | 0.0 | | | 0 | 0.0 | |
| 75-79 | 0 | | 0.0 | 0 | 0.0 | | | 0 | 0.0 | |
| Total | 18 | | 100.0 | 5 | 100.0 | | | 8 | 100.0 | |
| Location by state and territory | | | | | | | | | | |
| NSW | 3 | | 16.7 | 0 | 0.0 | | | 1 | 12.5 | |
| Vic | 3 | | 16.7 | 1 | 20.0 | | | 1 | 12.5 | |
| Qld | 3 | | 16.7 | 3 | 60.0 | | | 1 | 12.5 | |
| WA | 3 | | 16.7 | 1 | 20.0 | | | 2 | 25.0 | |
| SA | 3 | | 16.7 | 0 | 0.0 | | | 2 | 25.0 | |
| Tas^d^ | 1 | | 5.6 | 0 | 0.0 | | | 1 | 12.5 | |
| NT^d^ | 1 | | 5.6 | 0 | 0.0 | | | 0 | 0.0 | |
| ACT^d^ | 1 | | 5.6 | 0 | 0.0 | | | 0 | 0.0 | |
| Total | 18 | | 100.0 | 5 | 100.0 | | | 8 | 100.0 | |

Acronyms and abbreviations: ACT - Australian Capital Territory, NSW - New South Wales,

NT - Northern Territory, Qld -Queensland, SA - South Australia, Tas - Tasmania, Vic - Victoria,

WA - Western Australia.

^a^ includes ATAPS administering agency staff of Medicare Locals and subcontracted provider agencies

^b^ includes eligible ATAPS referrers

^c^ includes eligible mental health professionals delivering ATAPS

^d^ One Medicare Local operated in each of these States and Territories at the time.
